# Thermally Regulated Curing–Degradation Windows of Epoxidized Soybean Oil-Based Epoxy–Anhydride Liquid Plugs for Sustainable High-Temperature Sealing

**DOI:** 10.3390/molecules31122097

**Published:** 2026-06-15

**Authors:** Yuexin Tian, Yintao Liu, Haifeng Dong, Guodong Zhang, Biao Su, Xiaofeng Liu, Xiangjun Liu

**Affiliations:** 1 Petroleum Engineering Technology Institute of Southwest Petroleum Branch, SINOPEC, Deyang 618000, China; 2 State Key Laboratory of Oil and Gas Reservoir Geology and Exploitation, Southwest Petroleum University, Chengdu 610500, China

**Keywords:** epoxidized soybean oil, epoxy–anhydride network, liquid plug, thermally regulated curing, post-service degradation, temporary sealing

## Abstract

High-temperature temporary sealing operations require liquid plug materials that can be placed as low-viscosity precursors, converted into mechanically stable networks under reservoir temperature, and subsequently removed after service. Existing epoxy-based sealing systems generally provide high post-curing strength, but the coordination among pumpability, thermally triggered curing, and post-service degradability remains insufficiently addressed. In this work, an epoxidized soybean oil (ESO)-modified epoxy–anhydride liquid plug was designed to regulate these sequential stages within a single material system. The precursor formulation, rheological transition, curing kinetics, mechanical response, network structure, and degradation behavior were evaluated using viscosity monitoring, curing-time tests, DSC, compression testing, DMA, gel fraction and swelling measurements, FTIR, and high-temperature degradation experiments. The optimized precursor exhibited an initial viscosity of 65.4 ± 2.1 mPa·s, remaining below the pumpability threshold of 100 mPa·s before curing. Its curing time was adjustable within 1–10 h at 120–140 °C through temperature and initiator regulation. ESO incorporation produced a non-monotonic mechanical response, with the optimized network reaching a compressive strength of 112.5 ± 3.5 MPa and an elastic modulus of 142.50 ± 5.26 MPa. FTIR and thermal–mechanical analyses supported the formation of an ester-rich epoxy–anhydride network containing both rigid epoxy-derived segments and ESO-derived flexible chains. In the post-service stage, degradation was strongly temperature dependent, with the characteristic unsealing time decreasing from 84 h at 120 °C to 24 h at 130 °C and 18 h at 140 °C. The combined results define a coupled curing–degradation window in which pumpable placement, thermal network formation, load-bearing sealing, and controlled unsealing are temporally separated but structurally connected.

## 1. Introduction

High-temperature temporary sealing has become an important technical requirement in wellbore isolation, fracture control, water shutoff, lost-circulation control, and temporary plugging operations, where sealing materials are expected to be injected as low-viscosity fluids, develop sufficient pressure-bearing capacity after placement, and be removed after service without causing persistent formation damage. Conventional temporary plugging systems, including particulate bridging agents, polymer gels, and degradable gel plugs, have been widely explored for fractured reservoirs and heterogeneous flow channels. These systems can provide temporary flow diversion or leakage control, but their performance is often constrained by the balance between placement feasibility, mechanical stability, temperature resistance, and post-treatment removability. Recent studies on degradable gels, self-degradable rubber plugs, and optimized temporary plugging layers have shown that degradation time, pressure-bearing capacity, and structural integrity should be considered together rather than as isolated performance indexes [1,2,3,4,5,6]. Field-oriented studies on temporary plugging and diversion in fractured or tight reservoirs have also emphasized that plugging materials must be evaluated not only by initial blocking efficiency, but also by their placement behavior, structural stability, and post-treatment removability [7,8]. This requirement is particularly relevant to temporary sealing materials used under high-temperature conditions, where premature curing may impair placement, whereas insufficient network formation may reduce sealing reliability.

Epoxy-based sealing materials provide a promising route for constructing high-strength temporary plugs because epoxy networks generally exhibit strong adhesion, high compressive strength, chemical resistance, and tunable curing behavior. Low-viscosity epoxy resin sealing agents have been reported to enter fine cracks and subsequently form rigid blocking barriers, while epoxy/anhydride systems can show adjustable curing behavior and degradability under selected chemical conditions [9]. Recent advances in high-performance epoxy vitrimers and dynamic covalent epoxy networks have demonstrated improved thermal resilience and network adaptability under cyclic thermal or mechanical loading, offering guidance for sealing material design [10,11,12]. More recently, epoxidized soybean oil (ESO)-based plugging agents have also been investigated as renewable resin systems for casing-damage treatment and crack plugging, indicating the potential of bio-based epoxy chemistry in oilfield sealing applications [13]. Some studies additionally explored hybrid bio-based epoxy systems incorporating lignin or tannin derivatives to enhance thermo-mechanical properties while maintaining degradability [14,15]. Compared with conventional polymer gels, epoxy-based liquid plugs can provide higher mechanical integrity after curing, but their use in temporary sealing is still limited by two coupled issues. First, the precursor must remain sufficiently fluid during mixing and placement, which requires the curing reaction to be delayed or regulated before the material reaches the target zone. Second, the cured network should not behave as a permanent resin barrier; it must undergo controlled structural disassembly after the sealing task is completed. Existing epoxy sealing studies have mainly focused on injection, mechanical strength, plugging pressure, or degradation behavior as separate performance indexes, while the time-programmed relationship among pumpability, thermal curing, load-bearing sealing, and post-service unsealing remains insufficiently defined [9,13]. Recent studies on epoxy network design have further shown that degradable, reprocessable, or dynamically adaptable epoxy architectures can improve sustainability and structural tunability, but these concepts have not yet been sufficiently translated into liquid-plug systems requiring staged injection, curing, sealing, and unsealing [16,17].

Bio-based epoxy systems derived from epoxidized vegetable oils have attracted increasing attention because they can introduce renewable carbon sources, flexible aliphatic chains, and reactive epoxy groups into thermosetting networks. ESO is a representative bio-based epoxy compound that can participate in curing reactions while improving the flexibility and sustainability of epoxy networks. Recent studies have reported ESO-based thermosets cured with tannic acid, anhydrides, lignin-derived components, vanillin-derived hardeners, and dynamic covalent chemistries, demonstrating that ESO can be used to tune thermal, mechanical, anti-corrosion, reprocessable, and degradable properties of bio-based epoxy materials [18,19,20,21,22,23,24,25,26,27]. In addition, life cycle and sustainability assessments of epoxy systems increasingly emphasize the importance of renewable feedstocks and environment-friendly processing [28,29]. In parallel, DSC-based curing-kinetic studies of bio-based epoxy and ESO-containing systems have shown that curing temperature, heating rate, and reaction conversion are closely coupled, providing a useful basis for analyzing thermally regulated epoxy–anhydride network formation [30,31,32,33,34]. In epoxy–anhydride systems, ring-opening reactions generate ester-containing crosslinked networks, which can provide mechanical integrity after curing and, under appropriate chemical environments, potential sites for later network disassembly. This structural feature makes ESO-modified epoxy–anhydride systems attractive for temporary sealing materials, because the same ester-rich network that supports strength after curing may also provide a chemical basis for post-service degradation.

Despite these advances, most bio-based epoxy studies have focused on thermoset synthesis, curing kinetics, thermal-mechanical behavior, degradability, or recyclability [16,17,30,31,32,33,34], whereas fewer studies have considered how these material properties should be coordinated as an operational time window for temporary high-temperature sealing. Recent works on multi-functional transient networks and dynamic networks have offered frameworks for time-dependent performance optimization, which could be leveraged to define operational windows for injection, service, and disassembly [35,36]. For a liquid plug, the key question is not only whether a cured network can be formed, but whether the material can pass through three sequential states in a controllable way: a pumpable precursor before curing, a load-bearing solid-like plug after thermal activation, and a degradable network after service. In this context, three characteristic times are critical: pumpable time (*t*_p_), curing time (*t*_c_), and post-service unsealing time (*t*_d_). A rational material design should not maximize curing rate and degradation rate independently, but should temporally separate network formation and network disassembly. However, this coupled *t*_p_–*t*_c_–*t*_d_ framework has rarely been used to describe ESO-based epoxy–anhydride liquid plugs.

Our previous work developed an ESO-based semi-liquid gel material for wellbore sealing and separately clarified its interfacial reinforcement and degradation-related behavior [37,38,39]. These studies demonstrated the feasibility of ESO-modified epoxy systems for sealing applications, but they did not explicitly establish a unified curing–degradation window that links precursor pumpability, thermally triggered curing kinetics, network stiffness, ester-rich structure formation, and post-service mechanical failure. Different from previously reported ESO-based plugging agents that mainly emphasized material preparation, crack-plugging ability, pressure-bearing performance, or degradability as individual performance indexes, the present study focuses on the time-programmed transformation of an ESO-based epoxy–anhydride liquid plug throughout its complete service cycle. The fundamental novelty of this work lies in defining ESO not only as a bio-based modifier for plugging materials, but also as a reactive structural regulator that participates in ester-rich network formation and enables the coordinated transition from a pumpable precursor to a load-bearing cured plug and finally to a degradable post-service residue. Building on this material platform, the present study focuses on the molecular and time-window regulation of an ESO-based epoxy–anhydride liquid plug. Rheological measurements, curing-time evaluation, DSC, compression testing, DMA, gel fraction and swelling analysis, FTIR, high-temperature degradation tests, and residual-strength measurements were combined to construct a coupled *t*_p_–*t*_c_–*t*_d_ model. This model experimentally integrates pumpable time (t_p_), curing time (t_c_), and post-service unsealing time (t_d_), thereby distinguishing the present study from previous ESO-based plugging systems in which injection, curing, sealing, and degradation were not quantitatively connected within one operational window. The objective is to clarify how ESO incorporation and thermal activation regulate network formation and subsequent disassembly, thereby providing a sustainable temporary sealing strategy that integrates injectability, high-temperature curing, mechanical integrity, and controlled post-service unsealing within one bio-based epoxy–anhydride material system.

## 2. Results and Discussion

### 2.1. Overall Curing–Sealing Performance of the Optimized ESO-Based Epoxy–Anhydride Liquid Plug

**Figure 1:**
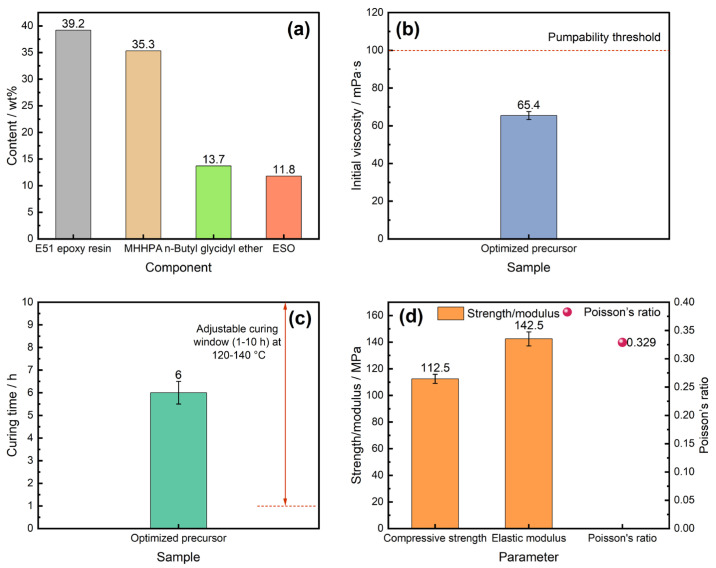
Overall curing–sealing performance of the optimized ESO-based epoxy–anhydride liquid plug: (**a**) optimized component composition; (**b**) initial viscosity and pumpability threshold; (**c**) representative curing time and adjustable curing window; (**d**) compressive strength, elastic modulus, and Poisson’s ratio of the cured plug. Data in (**b**–**d**) are presented as mean ± SD, *n* = 3.

The overall curing–sealing performance of the optimized ESO-based epoxy–anhydride liquid plug was first evaluated, as shown in Figure 1. The formulation consisted of 39.2 wt% E51 epoxy resin, 35.3 wt% MHHPA, 13.7 wt% n-butyl glycidyl ether, and 11.8 wt% ESO (Figure 1a). This exact formulation was selected as the optimized composition based on preliminary formulation screening, in which precursor viscosity, curing-time controllability, cured mechanical strength, and post-service degradability were jointly considered. E51 and MHHPA were maintained as the main epoxy–anhydride reactive framework to ensure sufficient crosslinking and load-bearing capacity after curing. n-Butyl glycidyl ether was introduced at an appropriate fraction to reduce precursor viscosity and improve pumpable placement, whereas ESO was incorporated as a bio-based reactive modifier to regulate network flexibility and introduce ester-rich degradable segments. E51 and MHHPA formed the main epoxy–anhydride reactive framework, while n-butyl glycidyl ether and ESO were introduced to regulate precursor flowability and network flexibility. Among the tested formulations, this composition provided the most balanced performance combination, including an initial viscosity below the pumpability threshold, an adjustable curing window, adequate compressive strength, and controlled post-service degradation. This composition was therefore used as the optimized formulation for subsequent pumpability, curing, mechanical, and post-service degradation analyses.

As shown in Figure 1b, the optimized precursor exhibited an initial viscosity of 65.4 ± 2.1 mPa·s, which remained below the pumpability threshold of 100 mPa·s adopted in this work. This low-viscosity state is important for a liquid plug precursor because the material must remain mobile during mixing and placement before thermal curing becomes dominant. In parallel, the representative curing time was 6.0 ± 0.5 h, and the curing interval could be adjusted within 1–10 h at 120–140 °C by regulating the initiator dosage (Figure 1c). Compared with a fixed gelation time, this adjustable curing range provides a more practical processing window for temporary high-temperature sealing, where the material needs to remain injectable during placement and then develop a continuous network after reaching the target zone.

The cured plug also retained adequate mechanical integrity after formulation adjustment. As summarized in Figure 1d, the compressive strength, apparent elastic modulus, and Poisson’s ratio were 112.5 ± 3.5 MPa, 142.50 ± 5.26 MPa, and 0.329 ± 0.012, respectively. These values indicate that the viscosity reduction and bio-based modification did not compromise the load-bearing capacity of the cured network. The reactive dilution effect of n-butyl glycidyl ether and the epoxy-containing flexible aliphatic segments introduced by ESO allowed the precursor to maintain injectability before curing while preserving the stiffness required for pressure-bearing sealing after thermal network formation.

Figure 1 provides the initial performance profile of the optimized ESO-based epoxy–anhydride liquid plug. The precursor satisfies the basic requirements for the first two stages of a curing–degradation window: pumpable placement and thermally triggered network formation. The following sections further examine the rheological transition, curing-time regulation, network formation, mechanical response, and post-service degradation behavior of this optimized system.

Compared with previous studies that mainly reported individual indexes such as initial viscosity, plugging strength, curing time, or degradation behavior, the significance of Figure 1 is that these properties are organized as sequential requirements for a temporary liquid plug. The optimized formulation did not simply provide a high-strength cured material; it simultaneously combined low precursor viscosity, adjustable curing time, and load-bearing mechanical integrity. This performance combination provides the experimental basis for distinguishing the present ESO-based epoxy–anhydride liquid plug from conventional epoxy sealing agents or ESO-based plugging materials evaluated only by isolated performance parameters.

### 2.2. Thermally Regulated Pumpability and Curing Kinetics

**Figure 2:**
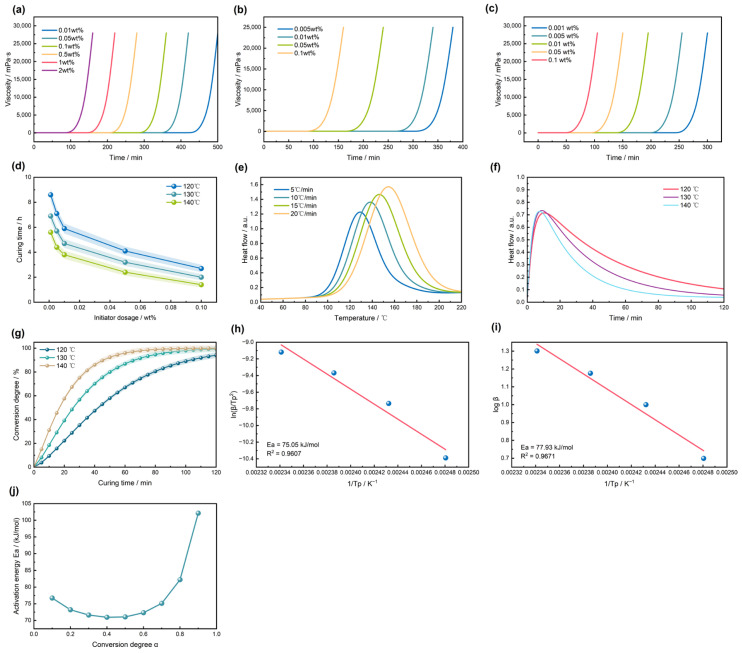
Thermally regulated pumpability and curing kinetics of the optimized ESO-based epoxy–anhydride liquid plug: (**a**–**c**) viscosity–time profiles at 120, 130, and 140 °C under different initiator dosages; (**d**) curing-time variation as a function of temperature and initiator dosage; (**e**) non-isothermal DSC curves at different heating rates; (**f**) isothermal DSC curves at 120, 130, and 140 °C; (**g**) conversion degree as a function of curing time. (**h**) Kissinger plot for apparent activation-energy calculation; (**i**) Ozawa plot for activation-energy verification; (**j**) conversion-dependent activation energy obtained from Friedman analysis. Data in (**d**,**g**) are presented as mean ± SD, *n* = 3.

The pumpability and curing behavior of the optimized ESO-based epoxy–anhydride liquid plug were systematically evaluated under high-temperature conditions, and the results are presented in Figure 2. As shown in Figure 2a–c, the viscosity–time profiles obtained at 120, 130, and 140 °C displayed a typical delayed-growth pattern rather than a continuous linear increase. In all cases, the precursor first maintained a relatively low viscosity and then underwent a sharp viscosity rise after a temperature- and initiator-dependent induction period. This transition marks the onset of rapid network formation induced by the thermally activated epoxy–anhydride curing reaction.

At 120 °C, the precursor retained a viscosity below the pumpability threshold of 100 mPa·s for a comparatively long period when the initiator dosage was controlled within 0.01–0.05 wt% (Figure 2a). This range provided sufficient time for precursor preparation, placement, and positional adjustment before substantial viscosity growth occurred. When the temperature increased to 130 °C, the suitable initiator range shifted to 0.005–0.05 wt% (Figure 2b), suggesting that less catalytic activation was required to achieve a comparable curing response. At 140 °C, the precursor became more sensitive to initiator dosage, and the effective low-viscosity region was narrowed to 0.001–0.01 wt% (Figure 2c). This contraction of the pumpability window indicates that thermal acceleration becomes progressively dominant at elevated temperature, and excessive initiator loading may markedly shorten the available operational interval.

The curing-time variation further supports this thermally regulated behavior. As summarized in Figure 2d, increasing either temperature or initiator dosage shortened the curing time. When the initiator dosage remained below 0.05 wt%, the precursor still maintained a curing time longer than 4 h within the 120–140 °C range, which is favorable for temporary sealing applications requiring sufficient placement time prior to in situ curing. These results show that pumpability and curing are intrinsically coupled rather than independent process variables, because both are governed by the combined effects of thermal activation and initiator-assisted epoxy–anhydride reaction.

To further clarify that the abrupt viscosity increase was associated with chemical curing rather than simple physical thickening, DSC analysis was incorporated into the same evaluation framework. The non-isothermal DSC curves in Figure 2e showed a distinct exothermic curing peak assigned to epoxy–anhydride ring-opening and subsequent esterification reactions. As the heating rate increased from 5 to 20 °C/min, the exothermic peak shifted toward higher temperature, reflecting the expected kinetic delay under continuous heating. The peak temperature increased from 130 °C at 5 °C/min to 138, 146, and 154 °C at 10, 15, and 20 °C/min, respectively. This behavior indicates that the system requires a higher apparent temperature to reach a comparable extent of conversion when the heating program is accelerated.

The isothermal DSC results shown in Figure 2f further confirmed the temperature sensitivity of the curing process. At 120, 130, and 140 °C, the exothermic heat flow gradually decayed with time, but the decay became significantly faster as temperature increased. This trend means that the reactive epoxy and anhydride groups were consumed more rapidly at higher temperature. The corresponding conversion curves in Figure 2g show that the precursor reached the same conversion level within a shorter time at elevated temperature, which is consistent with the viscosity-growth behavior in Figure 2a–c and the curing-time trend in Figure 2d.

To provide a more quantitative curing-kinetics interpretation, the non-isothermal DSC data were further analyzed using Kissinger, Ozawa, and Friedman methods. The exothermic peak temperatures extracted from Figure 2e increased from 130 °C at 5 °C/min to 138, 146, and 154 °C at 10, 15, and 20 °C/min, respectively. As shown in Figure 2h,i, the Kissinger and Ozawa plots gave apparent activation energies of 75.05 and 77.93 kJ/mol, respectively, confirming that the epoxy–anhydride curing reaction was strongly governed by thermal activation.

Friedman analysis further showed that the apparent activation energy varied with conversion degree rather than remaining constant throughout curing (Figure 2j). The Ea value decreased slightly from 76.72 kJ/mol at α = 0.1 to 70.94 kJ/mol at α = 0.4, remained relatively stable at intermediate conversion, and then increased markedly to 102.12 kJ/mol at α = 0.9. This trend suggests that the early-to-middle curing stage was mainly controlled by epoxy–anhydride ring opening and esterification, whereas the high-conversion stage was increasingly affected by restricted molecular mobility and diffusion limitation in the growing crosslinked network. These kinetic results provide quantitative support for the temperature-dependent shortening of curing time and for defining *t*_c_ as a chemically meaningful parameter in the coupled *t*_p_–*t*_c_–*t*_d_ window model.

The viscosity, curing-time, and DSC results in Figure 2a–g jointly define the thermally regulated pumpability–curing relationship of the ESO-based epoxy–anhydride precursor. A lower initiator dosage helps preserve a sufficiently long pumpable period, whereas elevated temperature accelerates network formation and shortens the transition from liquid precursor to solid-like cured structure. In this sense, the pumpable time (*t*_p_) and curing time (*t*_c_) are both governed by the same thermally activated chemical process, but they describe different stages of material response. This relationship provides the kinetic basis for the coupled curing–degradation window model discussed in Section 2.6.

### 2.3. ESO-Mediated Network Density, Thermomechanical Response, and Stiffness–Flexibility Balance

**Figure 3:**
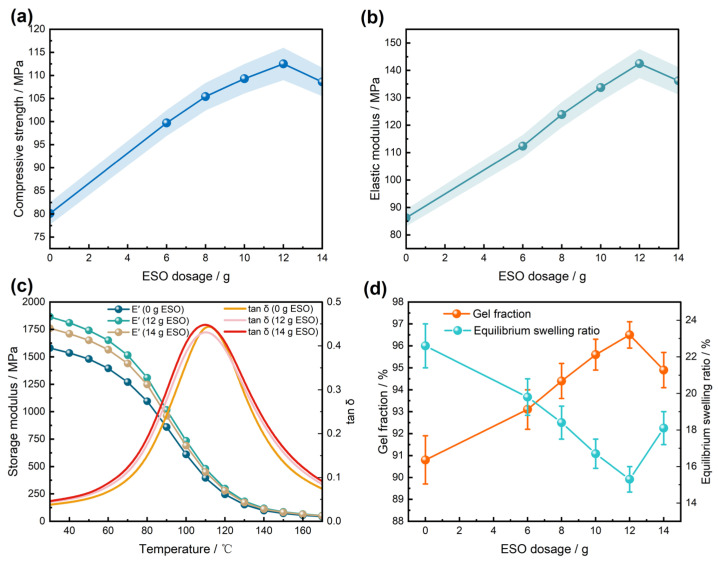
ESO-mediated network density, thermomechanical response, and stiffness–flexibility balance of the cured epoxy–anhydride liquid plug: (**a**) compressive strength as a function of ESO dosage; (**b**) elastic modulus as a function of ESO dosage; (**c**) representative DMA results showing storage modulus and tan δ behavior; (**d**) gel fraction and equilibrium swelling ratio as a function of ESO dosage. Data in (**a**,**b**,**d**) are presented as mean ± SD, *n* = 3.

To clarify how ESO regulates the mechanical response of the cured epoxy–anhydride liquid plug, the effects of ESO dosage on compressive strength, elastic modulus, thermomechanical behavior, and network integrity were evaluated, as summarized in Figure 3.

As shown in Figure 3a, the compressive strength increased from 80.1 MPa for the unmodified sample to 99.7, 105.4, 109.3, and 112.5 MPa when the ESO dosage increased to 6, 8, 10, and 12 g, respectively, and then slightly decreased to 108.6 MPa at 14 g. A similar trend was observed for the elastic modulus (Figure 3b), which increased from 86.24 MPa without ESO to 142.50 MPa at 12 g ESO, followed by a moderate decrease to 136.18 MPa at 14 g. These results show that ESO did not simply act as a softening modifier. Within a proper dosage range, it improved both load-bearing strength and stiffness, whereas excessive ESO weakened the reinforcing effect.

The DMA results further support this trend from a thermomechanical perspective. As shown in Figure 3c, the storage modulus increased progressively from the unmodified formulation to the sample containing 12 g ESO, indicating that the cured network became mechanically more effective in resisting deformation. Meanwhile, the tan δ peak became slightly broader after ESO incorporation, and the corresponding relaxation peak shifted only modestly toward lower temperature. This behavior suggests that ESO introduced flexible aliphatic segments into the network and improved energy-dissipation capability, but did not convert the cured system into a highly plasticized structure. In other words, the ESO-derived flexible chains increased segmental mobility to a limited extent, while the epoxy–anhydride crosslinked framework still preserved sufficient rigidity.

The gel fraction and equilibrium swelling ratio provide additional evidence for changes in network integrity. As shown in Figure 3d, the gel fraction increased from 90.8% in the ESO-free sample to 96.5% at 12 g ESO, whereas the equilibrium swelling ratio decreased from 22.6% to 15.3% over the same dosage range. This combination points to a more integrated and compact crosslinked structure after moderate ESO incorporation. At 14 g ESO, however, the gel fraction declined slightly to 94.9%, and the swelling ratio increased to 18.1%, suggesting that the network became somewhat less compact when excessive flexible segments were introduced. These structural changes are consistent with the mechanical results and help explain why the strengthening effect of ESO reached an optimum rather than increasing indefinitely. In this context, the gel fraction and swelling results provide an indirect but useful indication of the apparent network integrity and crosslinking compactness. A higher gel fraction reflects a lower soluble fraction after solvent extraction, whereas a lower equilibrium swelling ratio indicates stronger restriction of solvent penetration by a more compact network. Therefore, the increase in gel fraction from 90.8% to 96.5% and the concurrent decrease in swelling ratio from 22.6% to 15.3% at 12 g ESO suggest that moderate ESO incorporation enhanced the effective crosslinked network rather than simply diluting the epoxy–anhydride matrix. This interpretation is further supported by the DMA results, where the optimized ESO-containing sample showed higher storage modulus and a broadened tan δ response, indicating improved stiffness retention with limited segmental relaxation.

The mechanical, thermomechanical, and network-integrity results consistently support the role of ESO as a reactive bio-based regulator rather than an inert diluent. At moderate dosage, its epoxy-containing flexible chains can be incorporated into the curing network, leading to a more balanced combination of strength, stiffness, and deformability. At excessive dosage, the growing fraction of flexible aliphatic segments begins to dilute the effective rigidity of the load-bearing framework. Therefore, the formulation containing about 12 g ESO provides the most favorable stiffness–flexibility balance among the tested samples, and this dosage was taken as the representative composition for the subsequent FTIR and degradation analyses. This result also explains why the 12 g ESO formulation was selected as the optimized network composition for the overall curing–sealing performance evaluation in Section 2.1.

### 2.4. FTIR Evidence for Ester-Rich Network Formation and Reactive ESO Incorporation

**Figure 4:**
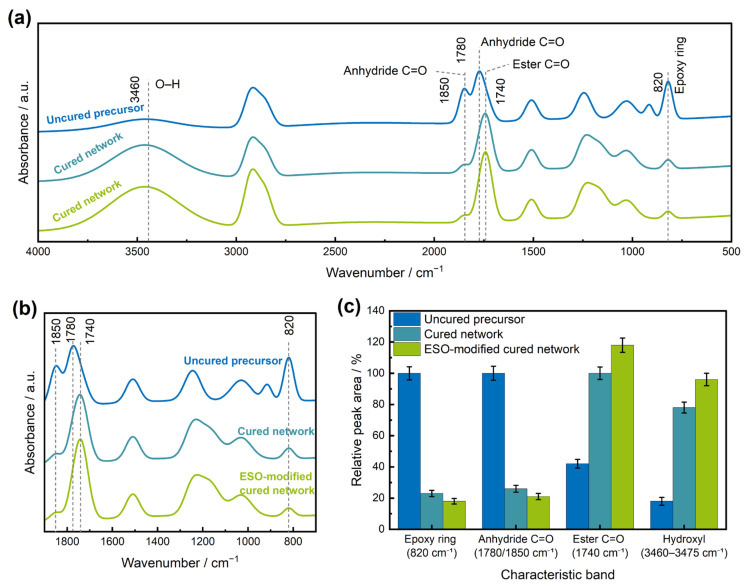
FTIR spectra of the ESO-based epoxy–anhydride liquid plug before and after thermal curing: (**a**) comparison of uncured precursor and cured samples; (**b**) enlarged region showing the variation in epoxy, anhydride, ester, and hydroxyl characteristic bands; (**c**) relative peak-area changes in epoxy and ester-related absorptions during curing. Data in (**c**) are presented as mean ± SD, *n* = 3.

FTIR analysis was conducted to verify the chemical evolution of the ESO-based epoxy–anhydride network during thermal curing, as shown in Figure 4. The uncured precursor exhibited characteristic absorption bands associated with epoxy and anhydride groups, while the cured samples displayed marked spectral changes after exposure to the selected curing conditions. The absorption band of the epoxy ring near 820 cm^−1^ weakened substantially after curing, and the anhydride-related carbonyl bands around 1780 and 1850 cm^−1^ were also greatly reduced. These changes are consistent with the ring-opening reaction between epoxy and anhydride groups during network formation.

A prominent ester carbonyl band appeared near 1740 cm^−1^ in the cured samples, accompanied by a broad hydroxyl absorption region around 3460–3475 cm^−1^. The formation of ester linkages agrees with the expected epoxy–anhydride curing pathway, in which anhydride opening produces ester-containing crosslinks and hydroxyl groups. Compared with the cured network without ESO, the ESO-modified cured network showed stronger hydroxyl-related absorption and more evident ester-rich spectral features. This difference suggests that ESO-derived epoxy groups were involved in the curing reaction rather than remaining as an inactive liquid modifier. This spectral evolution provides chemical evidence for the incorporation of ESO-derived reactive segments into the epoxy–anhydride network. If ESO mainly acted as an unreacted plasticizing phase, the cured system would be expected to show weaker network-related changes and less consistent improvement in gel fraction, swelling resistance, and thermomechanical stiffness. In contrast, the simultaneous reduction in epoxy/anhydride characteristic bands, enhancement of ester-related absorption, increased gel fraction, reduced swelling ratio, and improved storage modulus support the view that ESO participated in network formation and contributed to the optimized crosslinking structure.

The spectral changes are also aligned with the mechanical trend discussed in Section 2.3. The incorporation of ESO did not weaken the network within the optimized dosage range; instead, the simultaneous growth of ester and hydroxyl features points to a more integrated network structure containing both rigid E51-derived segments and flexible soybean-oil-derived chains. Such a structure provides a plausible chemical basis for the improved compressive strength and modulus observed at moderate ESO contents. However, the FTIR spectra mainly confirm functional-group conversion and cannot independently quantify crosslink density; therefore, the stiffness–flexibility balance is further interpreted together with the mechanical data.

The FTIR results support the formation of an ester-rich epoxy–anhydride network and provide chemical evidence for the reactive incorporation of ESO during thermal curing. This chemical evidence helps connect the formulation design with the subsequent performance window: the same ester-containing network that contributes to mechanical integrity after curing may also provide chemically responsive sites for post-service degradation.

### 2.5. Temperature-Activated Degradation and Residual Mechanical Failure of Ester-Containing Networks

**Figure 5:**
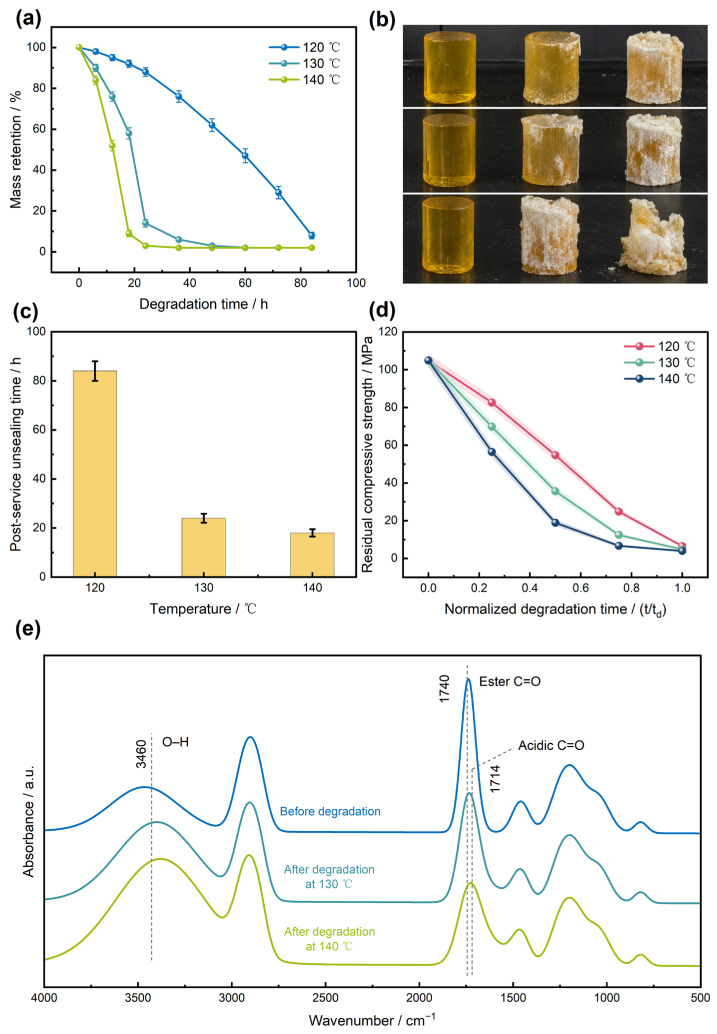
Temperature-activated degradation and residual mechanical failure of the ester-containing ESO-based epoxy–anhydride liquid plug: (**a**) mass-retention profiles at 120, 130, and 140 °C; (**b**) representative macroscopic evolution of the cured plug during degradation; (**c**) characteristic post-service unsealing time, *t*_d_, at different temperatures; (**d**) residual compressive strength during degradation. (**e**) FTIR spectra before and after degradation showing weakening of ester-related bands. Data in (**a**,**c**,**d**) are presented as mean ± SD, *n* = 3.

The post-service degradation behavior of the cured ESO-based epoxy–anhydride liquid plug was evaluated at 120, 130, and 140 °C to determine whether the ester-containing network could undergo thermally activated disassembly after temporary sealing, as shown in Figure 5.

As shown in Figure 5a, the mass retention decreased progressively with degradation time at all tested temperatures, but the degradation rate was strongly temperature-dependent. At 120 °C, the decrease was relatively gradual, and the cured plug retained part of its macroscopic integrity during the early degradation stage. A nearly disintegrated state was reached after approximately 84 h. When the temperature increased to 130 °C, the mass-loss process was markedly accelerated, and the characteristic structural collapse occurred within about 24 h. At 140 °C, the degradation process became even faster, and the loss of macroscopic integrity was shortened to approximately 18 h.

The mass-retention profiles did not follow a simple linear decay behavior. During the initial stage, the samples mainly exhibited swelling and softening, while the overall cylindrical morphology was still partly preserved. With prolonged exposure, the mass-retention curves entered a more rapid decline stage, which is likely associated with progressive network loosening and cleavage of ester-containing segments. This interpretation is consistent with the FTIR results in Section 2.4, where the cured plug was shown to contain abundant ester linkages generated by epoxy–anhydride ring-opening and esterification reactions. The faster degradation at elevated temperature may therefore be related to enhanced molecular mobility, faster penetration of the degradation medium, and greater susceptibility of ester-rich regions to chemical attack.

The representative macroscopic evolution shown in Figure 5b further supports this degradation sequence. The original cured sample appeared as a translucent amber cylindrical body. As degradation proceeded, the sample gradually lost transparency, developed whitish eroded regions and surface peeling, and eventually transformed into a loose, partially collapsed residue. This morphological transition became progressively faster as the temperature increased from 120 to 140 °C, in agreement with the mass-retention trends in Figure 5a. These observations indicate that degradation involved not only chemical weakening of the crosslinked network but also physical loss of structural continuity.

To directly verify the chemical origin of network disassembly, FTIR spectra were collected before and after degradation, as shown in Figure 5e. The cured plug before degradation exhibited a distinct ester carbonyl absorption near 1740 cm^−1^, consistent with the ester-rich epoxy–anhydride network identified in Section 2.4. After degradation at 130 °C and 140 °C, this ester-related band became markedly weaker, while the broad hydroxyl/carboxyl-related absorption near 3460 cm^−1^ increased. These spectral changes provide direct chemical evidence that post-service degradation involved cleavage of ester-containing crosslinking segments and formation of hydroxyl- and carboxyl-containing degradation products, rather than being only a result of physical swelling or surface erosion.

The observed weakening of the ester-related absorption correlates with the accelerated mass loss (Figure 5a), macroscopic morphological collapse (Figure 5b), and rapid decrease in residual compressive strength (Figure 5d). This demonstrates that temperature-dependent degradation of the ester-rich network governs the post-service unsealing process, providing a molecular-level explanation for the observed td shortening and structural failure.

To verify whether the observed morphological collapse corresponded to a real loss of sealing capability, the residual compressive strength was measured at representative degradation stages, and the results are presented in Figure 5d. At 120 °C, the residual compressive strength decreased gradually from the initial optimized value of 112.5 MPa to a low level as degradation approached the characteristic unsealing time. In contrast, the strength loss was much faster at 130 and 140 °C. When the normalized degradation time reached 0.50, the residual compressive strength had already decreased to 35.6 MPa at 130 °C and 18.9 MPa at 140 °C, compared with 54.7 MPa at 120 °C. Near the corresponding $t_{d}$, all samples showed only very limited residual load-bearing capacity, indicating that the unsealing time represented not merely a visual collapse endpoint but also a mechanical–structural failure time.

The characteristic post-service unsealing time summarized in Figure 5c decreased from approximately 84 h at 120 °C to 24 h at 130 °C and 18 h at 140 °C. This marked reduction confirms that temperature acts as an effective trigger for post-service network disassembly, providing a feasible unsealing window for temporary high-temperature sealing applications. The agreement among mass-retention curves, macroscopic evolution, residual-strength decay, and FTIR evidence in Figure 5e demonstrates that the ESO-based epoxy–anhydride network undergoes controlled, temperature-activated degradation of ester-containing structures, enabling post-service removal while maintaining stability during the sealing period.

### 2.6. Coupled Curing–Degradation Window for Sustainable High-Temperature Temporary Sealing

**Figure 6:**
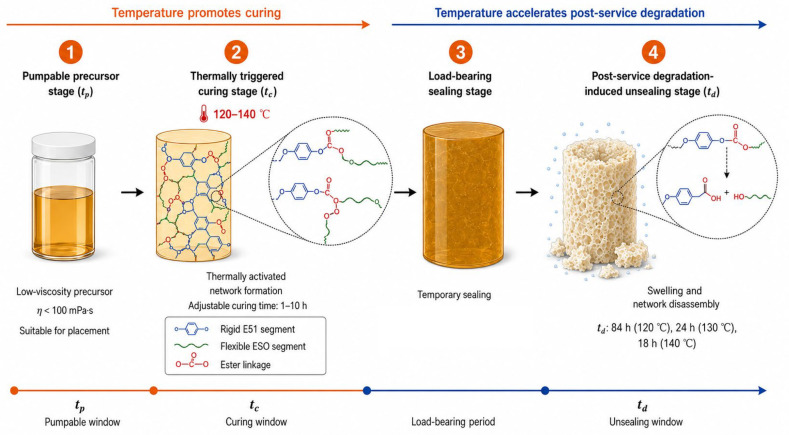
Schematic representation of the coupled curing–degradation window of the ESO-based epoxy–anhydride liquid plug: low-viscosity pumpable precursor stage (*t*_p_), thermally triggered network formation stage (*t*_c_), load-bearing sealing stage, and post-service degradation-induced unsealing stage (*t*_d_). The blue circles represent the degradation medium penetrating into the cured network during post-service unsealing.

The preceding results indicate that the ESO-based epoxy–anhydride liquid plug does not behave as a conventional permanently cured resin. Instead, its practical function depends on a staged material response: the precursor must remain injectable during placement, transform into a load-bearing network under reservoir temperature, and subsequently undergo controllable disassembly after service. To describe this time-programmed behavior, a coupled curing–degradation window was constructed by integrating three experimentally determined characteristic times: pumpable time (*t*_p_), curing time (*t*_c_), and post-service unsealing time (*t*_d_), as schematically summarized in Figure 6.

This *t*_p_–*t*_c_–*t*_d_ model differs from conventional curing or degradation studies because it does not treat curing kinetics, network formation, and degradation behavior as isolated material properties. Instead, it defines whether these processes are temporally compatible within one temporary sealing operation: *t*_p_ reflects the available placement interval before excessive viscosity growth, *t*_c_ represents the transition from a pumpable precursor to a mechanically stable sealing body, and td describes the post-service loss of both structural integrity and load-bearing capability. Therefore, the model provides an operation-oriented curing–degradation window rather than a single curing-rate or degradation-rate evaluation.

In this model, *t*_p_ represents the period during which the precursor viscosity remains below the pumpability threshold of 100 mPa·s. It was extracted from the viscosity–time profiles in Figure 2a–c. This parameter corresponds to the practical interval available for mixing, placement, and positional adjustment before extensive network formation. The *t*_c_ value describes the time required for the precursor to lose macroscopic flowability and form a continuous epoxy–anhydride network under thermal activation. It was determined from the curing-time measurements in Figure 2d and supported by the DSC curing behavior in Figure 2e–g. The viscosity-growth profiles and DSC curing curves in Section 2.2 jointly show that both *t*_p_ and *t*_c_ are governed by temperature-accelerated curing kinetics rather than by physical thickening alone. After curing, the network enters a load-bearing sealing stage, supported by the compressive strength, elastic modulus, DMA response, gel fraction, and swelling resistance discussed in Section 2.1 and Section 2.3. The final parameter, *t*_d_, represents the time required for the cured plug to lose macroscopic structural integrity and residual load-bearing capability in the degradation medium. It was obtained from the combined mass-retention, morphological evolution, and residual-strength results in Figure 5a–d. These three parameters therefore define the sequential transition from liquid precursor to cured sealing body and then to post-service disassembled residue.

Although the present *t*_p_–*t*_c_–*t*_d_ model was established from experimentally measured characteristic times, it also has predictive potential for formulation screening and operational-window selection. Once the relationships between temperature, initiator dosage, and tp/tc are obtained from viscosity-growth and curing-kinetic measurements, the model can be used to estimate whether a precursor formulation can retain sufficient pumpability before placement and complete network formation within the desired sealing period. Similarly, when the temperature-dependent td and residual-strength decay are determined for a given degradation medium, the model can help evaluate whether the cured plug is expected to lose structural integrity and load-bearing capability within an acceptable post-service unsealing time. Therefore, the predictive value of this model lies not in replacing detailed kinetic or field-scale validation, but in providing a semi-quantitative framework to connect formulation variables, thermal conditions, and service-stage requirements. Its current applicability is limited to the tested temperature range, initiator range, and degradation medium, while further calibration under broader reservoir conditions would improve its predictive robustness.

The temperature dependence observed in Section 2.2 and Section 2.5 suggests that temperature acts as a dual regulator in this system. During placement and curing, elevated temperature accelerates epoxy–anhydride ring-opening and shortens the curing interval. During post-service treatment, the same temperature increase promotes swelling, network loosening, and degradation of ester-containing structures. This dual role does not necessarily create a contradiction because curing and degradation occur in different service stages and under different chemical environments. In the absence of degradation medium, thermal activation mainly promotes network formation; after the degradation medium is introduced, the ester-rich network becomes susceptible to structural disassembly.

Figure 6 summarizes this coupled response. At the precursor stage, low viscosity enables placement before extensive network formation. During the curing stage, the precursor is converted into an ester-crosslinked network containing rigid E51-derived segments, flexible ESO-derived aliphatic chains, and ester linkages. This network provides the stiffness and compressive resistance needed for temporary high-temperature sealing. After the sealing operation, the degradation medium induces swelling and weakens ester-containing regions, leading to loss of network continuity and eventual unsealing. The residual-strength results in Section 2.5 further show that *t*_d_ is not only a morphological endpoint but also a mechanical-failure time, as the cured plug retains only limited load-bearing capacity near the unsealing stage.

A useful design implication of this model is that curing and degradation should not be optimized independently. Instead, network formation and network disassembly need to be separated in time. A suitable temporary liquid plug should provide a sufficiently long *t*_p_ for placement, a controllable *t*_c_ for in situ curing and strength build-up, and a post-service *t*_d_ that allows removal after the sealing task is completed. From this perspective, the ESO-based epoxy–anhydride network offers a sustainable temporary sealing strategy by combining bio-based reactive modification, thermally regulated curing kinetics, mechanical integrity, and chemically triggered post-service degradability within one material platform.

This distinction is important when compared with the existing literature, especially our previous ESO-based liquid-plug studies [37,38,39]. Those studies demonstrated the feasibility of ESO-modified systems for wellbore sealing and clarified interfacial reinforcement, diffusion-driven degradation, and silane-induced microstructure engineering as separate aspects. However, they did not establish a unified operational window that quantitatively connects precursor pumpability, thermally triggered curing, load-bearing sealing, and post-service unsealing. In contrast, the present work connects these outcomes into a single time-programmed framework by defining *t*_p_, *t*_c_, and *t*_d_ as linked characteristic times. The novelty therefore does not only arise from using ESO as a bio-based component, but from demonstrating how ESO-mediated ester-rich network formation can be coordinated with pumpable placement, thermal curing, mechanical sealing, and post-service degradation. This result-oriented comparison clarifies that the present system is designed as an operationally staged temporary sealing platform rather than a permanently cured resin or a single-function degradable plugging agent.

## 3. Materials and Methods

### 3.1. Materials

Epoxy resin E51 (industrial grade, epoxy equivalent weight: 184–194 g/eq) was supplied by Changzhou Runxiang Chemical Co., Ltd. (Changzhou, China) and used as the main epoxy matrix. Methylhexahydrophthalic anhydride (MHHPA, 98%) was purchased from Changzhou Runxiang Chemical Co., Ltd. (Changzhou, China) and used as the anhydride curing agent. n-Butyl glycidyl ether (industrial grade, purity ≥ 98%) was obtained from Changzhou Runxiang Chemical Co., Ltd. (Changzhou, China) and used as a reactive diluent to adjust the precursor viscosity. Epoxidized soybean oil (ESO, analytical grade, epoxy value: 6.0–6.5%) was purchased from Tianjin Kemiou Chemical Reagent Co., Ltd. (Tianjin, China) and introduced as a bio-based reactive modifier.

N,N-Dimethylbenzylamine (BDMA, 98%) was supplied by Shanghai Macklin Biochemical Co., Ltd. (Shanghai, China) and used as a tertiary amine initiator for the epoxy–anhydride curing reaction. γ-Valerolactone (GVL, 98%) was purchased from Shanghai Aladdin Biochemical Technology Co., Ltd. (Shanghai, China), and p-toluenesulfonic acid monohydrate (p-TSA, 98%) was obtained from Sinopharm Chemical Reagent Co., Ltd. (Shanghai, China). These two chemicals were used to prepare the degradation medium for post-service unsealing evaluation. Ethanol (analytical grade) was purchased from Chengdu Kelong Chemical Reagent Co., Ltd. (Chengdu, China) and used for rinsing degraded specimens. All reagents were used as received without further purification.

The optimized ESO-based epoxy–anhydride liquid plug formulation consisted of 39.2 wt% E51 epoxy resin, 35.3 wt% MHHPA, 13.7 wt% n-butyl glycidyl ether, and 11.8 wt% ESO. The BDMA dosage was adjusted according to the curing-time and rheological evaluation requirements. For degradation evaluation, the degradation medium was prepared by dissolving p-toluenesulfonic acid monohydrate in γ-valerolactone at a mass ratio of GVL/p-TSA = 95/5 (*w*/*w*). Cured specimens were then treated in this GVL/p-TSA degradation medium under selected high-temperature conditions.

### 3.2. Instruments

The main instruments used in this study included a C3003 analytical balance (Hangzhou Wante Weighing Instrument Co., Ltd., Hangzhou, China), an HH-SJ digital thermostatic magnetic oil bath (Changzhou Guoyu Instrument Manufacturing Co., Ltd., Changzhou, China), a DJ1C-60 high-torque mechanical stirrer (Jiangsu Jincheng Guosheng Laboratory Instrument Factory, Changzhou, China), and a DZF-6020 vacuum drying oven (Shanghai Yiheng Scientific Instrument Co., Ltd., Shanghai, China). These instruments were used for reagent weighing, precursor mixing, thermal treatment, degassing, drying, and sample conditioning.

Rheological measurements were performed using a DV-II rotational viscometer (AMETEK Brookfield, Middleboro, MA, USA). Curing-time tests were conducted in a thermostatic oil bath with a temperature accuracy of ±1 °C. Fourier transform infrared spectroscopy (FTIR) spectra were recorded using a Nicolet iS10 FTIR spectrometer (Thermo Fisher Scientific, Waltham, MA, USA). Differential scanning calorimetry (DSC) was performed using a DSC 214 Polyma instrument (NETZSCH-Gerätebau GmbH, Selb, Germany) under a nitrogen atmosphere. Dynamic mechanical analysis (DMA) was conducted using a DMA Q800 analyzer (TA Instruments, New Castle, DE, USA). The detailed analytical procedures for FTIR, DSC, and DMA are described in Section 3.10, Section 3.6, and Section 3.8, respectively.

Compression tests and residual mechanical-strength measurements were performed using a WDW-100E universal testing machine (Jinan New Era Testing Equipment Co., Ltd., Jinan, China). Cylindrical molds and PTFE molds were used for specimen preparation. Digital calipers were used to measure specimen dimensions before mechanical testing. High-temperature degradation experiments were carried out in sealed stainless-steel reactors placed in the DZF-6020 vacuum drying oven described above.

### 3.3. Preparation of ESO-Based Epoxy–Anhydride Liquid Plug Precursors

**Figure 7:**
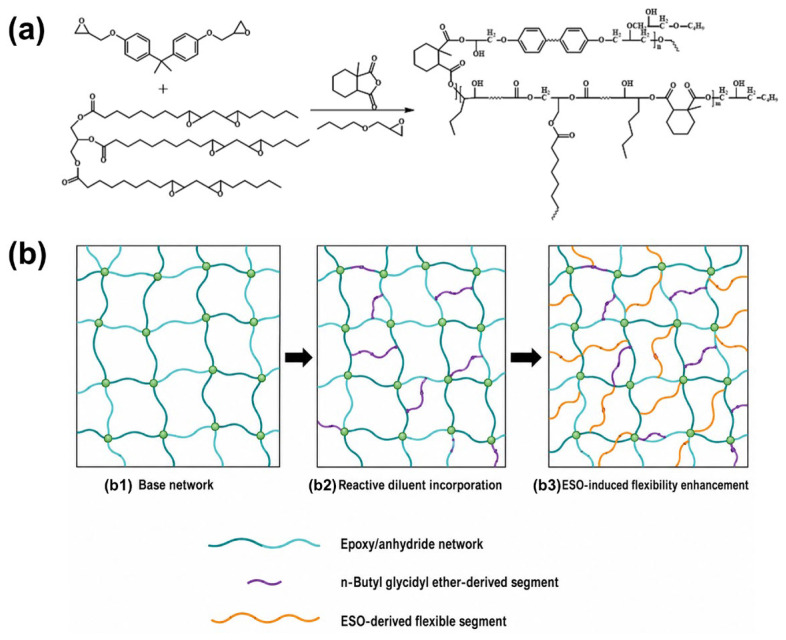
Reaction pathway and network evolution of the ESO-based epoxy–anhydride liquid plug precursor: (**a**) epoxy–anhydride ring-opening reaction among E51 epoxy resin, methylhexahydrophthalic anhydride (MHHPA), n-butyl glycidyl ether, and epoxidized soybean oil (ESO); (**b**) schematic network evolution, including (**b1**) base epoxy/anhydride network, (**b2**) reactive-diluent-incorporated network, and (**b3**) ESO-induced flexibility-enhanced network. The reaction-network concept was adapted from our previously reported ESO-based semi-liquid gel system [19].

The ESO-based epoxy–anhydride liquid plug precursor was formulated by incorporating epoxidized soybean oil (ESO) into an E51 epoxy–MHHPA reactive system. As shown in Figure 7, the optimized formulation consisted of 39.2 wt% E51 epoxy resin, 35.3 wt% methylhexahydrophthalic anhydride (MHHPA), 13.7 wt% n-butyl glycidyl ether, and 11.8 wt% ESO. E51 served as the main epoxy matrix, MHHPA acted as the anhydride curing agent, n-butyl glycidyl ether was used as a reactive diluent, and ESO was introduced as a bio-based reactive modifier. The scheme illustrates both the epoxy–anhydride ring-opening reaction and the corresponding network evolution induced by the reactive diluent and ESO-derived flexible segments.

Initially, the epoxy resin and ESO were weighed according to the target formulation and combined in a 50 mL glass beaker. The mixture was maintained at 60–80 °C under continuous magnetic stirring for 20 min to achieve homogeneous dispersion. Subsequently, the diluent n-butyl glycidyl ether was added gradually and stirred for an additional 10 min, followed by the slow incorporation of MHHPA, ensuring uniform mixing and minimizing premature ring-opening reactions. To preserve pumpability and control reaction onset, no catalyst was added at this stage.

The resulting homogeneous precursor mixture was then degassed under vacuum for 15 min to remove entrapped air bubbles, thereby improving network uniformity and avoiding defects during subsequent curing. The degassed liquid plug precursor was transferred into cylindrical molds (ϕ 25 mm × 50 mm) for thermal curing. Curing was performed in a constant-temperature oven at 120–140 °C, with curing times adjustable between 1 and 10 h depending on the desired crosslink density and intended service window, as established from preliminary kinetic analyses.

From a molecular perspective, the incorporation of ESO introduced flexible aliphatic chains with reactive epoxy groups that participate in the anhydride ring-opening polymerization. This dual function allowed the system to simultaneously maintain low viscosity for injection and to form a polyester-crosslinked network upon heating. The n-butyl glycidyl ether further enhanced miscibility and controlled the network density, contributing to a tunable balance between stiffness and flexibility. After curing, uniform semi-solid gel samples with high compressive strength and controlled degradation potential were obtained, serving as the foundation for subsequent evaluation of thermally regulated curing–degradation behavior.

### 3.4. Rheological Evaluation and Pumpability Window Determination

The rheological behavior of the ESO-based epoxy–anhydride liquid plug precursor was measured using a DV-II rotational viscometer (AMETEK Brookfield, Middleboro, MA, USA). For each test, 50 mL of freshly prepared precursor was transferred into the measuring cup and tested at a fixed spindle speed of 50 r/min. The apparent viscosity was recorded under isothermal conditions at 120, 130, and 140 °C to simulate the thermal range relevant to high-temperature sealing. Before testing, the measuring cup was preheated to the target temperature, and the precursor was loaded immediately after mixing to reduce the deviation caused by temperature lag.

To evaluate the influence of catalytic activation on flowability retention, N,N-dimethylbenzylamine was added at the designed dosage and mixed uniformly before viscosity recording. The viscosity–time curve was monitored until the precursor lost measurable flowability. The pumpability window was defined as the period during which the apparent viscosity remained below 100 mPa·s, and the corresponding time was recorded as tp. Each condition was tested three times, and the average value was used to construct the temperature-dependent pumpability profile.

### 3.5. Thermally Regulated Curing Time Measurement

The curing time of the ESO-based epoxy–anhydride liquid plug precursor was measured under isothermal conditions to evaluate its temperature-regulated curing behavior. Freshly prepared precursor samples were transferred into sealed glass vials immediately after mixing. The vials were then placed in a thermostatic oil bath at 120, 130, and 140 °C, respectively. For each temperature, N,N-dimethylbenzylamine was added at the designed dosage before curing to regulate the epoxy–anhydride ring-opening reaction.

The curing time was determined by an inversion method. At predetermined intervals, the vial was removed from the oil bath and inverted for visual observation. The sample was considered cured when no visible flow or surface deformation was observed after inversion. The time from the start of isothermal heating to this state was recorded as the curing time, *t*_c_. Each condition was tested in triplicate, and the average value was reported with a variation of ±0.5 h.

### 3.6. DSC Curing Analysis

Differential scanning calorimetry (DSC) was used to evaluate the thermally regulated curing behavior of the ESO-based epoxy–anhydride precursor. Freshly prepared precursor samples containing the designed dosage of N,N-dimethylbenzylamine were sealed in aluminum pans before testing. The sample mass was controlled within a consistent range for all DSC measurements, and the pans were sealed immediately after loading to minimize evaporation and pre-curing before thermal scanning. Non-isothermal DSC measurements were performed under a nitrogen atmosphere at heating rates of 5, 10, 15, and 20 °C/min over the temperature range of 40–220 °C. The curing exotherm and peak temperature were recorded to compare the influence of heating rate on the epoxy–anhydride curing process. The non-isothermal DSC data were further used for Kissinger, Ozawa, and Friedman kinetic analyses.

Isothermal DSC tests were further conducted at 120, 130, and 140 °C to match the temperature range used in rheological and curing-time measurements. The heat-flow signal was recorded as a function of time until the exothermic response approached a stable baseline. The relative curing conversion was calculated from the normalized cumulative heat release according to Equation (1):

(1) $$\alpha_{t}=\frac{\Delta H_{t}}{\Delta H_{\mathrm{total}}}\times 100\%$$

where *α*_t_ is the relative curing conversion at time t, Δ*H*_t_ is the cumulative heat release up to time t, and Δ*H*_total_ is the total heat release obtained from the complete curing process. Each isothermal condition was tested in triplicate, and the conversion data were reported as mean ± SD.

### 3.7. Mechanical Strength and Stiffness Evaluation

The mechanical strength and stiffness of the cured ESO-based epoxy–anhydride liquid plug were evaluated by uniaxial compression testing. Cylindrical specimens were prepared by pouring the degassed precursor into PTFE molds and curing under the selected temperature–time conditions. After curing, the specimens were demolded and conditioned at room temperature for 24 h before testing. The final specimen dimensions were 25 mm in diameter and 25 mm in height, and samples with visible bubbles, surface defects, or dimensional deviation were excluded.

Compression tests were performed using a WDW-100E universal testing machine (Jinan New Era Testing Equipment Co., Ltd., Jinan, China) at a loading rate of 1 mm/min. The compressive strength was calculated from the maximum load divided by the initial cross-sectional area of the specimen. The apparent compressive modulus was obtained from the initial linear region of the stress–strain curve to evaluate the stiffness evolution of the cured network. At least three parallel specimens were tested for each formulation or curing condition, and the results were reported as average values with standard deviations.

### 3.8. Dynamic Mechanical Analysis

Dynamic mechanical analysis (DMA) was performed to evaluate the thermomechanical response of cured ESO-based epoxy–anhydride specimens with different ESO dosages. Cured specimens were prepared under the selected curing conditions and cut into rectangular strips with similar dimensions before testing. Samples with visible defects, bubbles, or irregular edges were excluded to reduce testing deviation.

DMA measurements were conducted using the instrument described in Section 3.2. The samples were tested in temperature-sweep mode from 30 to 170 °C at a heating rate of 3 °C/min and a fixed frequency of 1 Hz. The storage modulus $E^{\prime }$ and loss factor tan δ were recorded as functions of temperature. The storage modulus was used to compare stiffness retention during heating, while tan δ was used to evaluate segmental relaxation and energy-dissipation behavior. Representative formulations, including the ESO-free sample, the optimized ESO-containing sample, and the excessive-ESO sample, were selected to clarify the relationship between ESO dosage and stiffness–flexibility balance.

### 3.9. Gel Fraction and Swelling Measurement

The gel fraction and equilibrium swelling ratio were measured to evaluate the network integrity and compactness of cured ESO-based epoxy–anhydride specimens. Cured samples with different ESO dosages were dried at 60 °C to constant mass, and the initial dry mass was recorded as $m_{1}$. The dried specimens were then immersed in tetrahydrofuran (THF) at room temperature for 24 h to remove soluble fractions and allow the network to reach swelling equilibrium.

After immersion, the swollen samples were removed, gently wiped with filter paper to eliminate surface solvent, and immediately weighed to obtain the swollen mass $m_{s}$. The samples were then dried again at 60 °C to constant mass and weighed as $m_{2}$. The gel fraction and equilibrium swelling ratio were calculated using Equations (2) and (3):

(2) $$G_{f}=\frac{m_{2}}{m_{1}}\times 100\%$$

(3) $$S_{e}=\frac{m_{s}-m_{2}}{m_{2}}\times 100\%$$

where *G_f_* is the gel fraction, *S_e_* is the equilibrium swelling ratio, *m*_1_ is the initial dry mass, *m*_s_ is the swollen mass after solvent immersion, and *m*_2_ is the dry mass after extraction and redrying. Each formulation was tested in triplicate, and the results were reported as mean ± SD.

### 3.10. FTIR Characterization of Network Formation

Fourier transform infrared spectroscopy (FTIR) was used to monitor the formation of the epoxy–anhydride network and the incorporation of ESO-derived reactive segments during curing, and the chemical changes before and after degradation. Uncured precursor samples and cured plug specimens obtained under selected curing conditions were collected for comparison. Degraded residues collected near the characteristic post-service unsealing stage were also analyzed to evaluate degradation-related chemical changes. The cured samples were dried at 60 °C to remove residual moisture and then ground into fine powders before testing.

FTIR spectra were recorded using a Nicolet iS10 spectrometer (Thermo Fisher Scientific, Waltham, MA, USA) in the range of 4000–500 cm^−1^ with a resolution of 4 cm^−1^. Each spectrum was obtained by accumulating 32 scans to improve the signal-to-noise ratio. The characteristic absorption bands associated with epoxy groups, anhydride groups, ester linkages, and hydroxyl groups and degradation-related carbonyl/hydroxyl changes were compared before and after curing. When required, peak areas were normalized against a relatively stable reference band to evaluate the relative variation in functional groups during network formation.

### 3.11. Degradation Behavior and Post-Service Unsealing Window

The degradation behavior of the cured ESO-based epoxy–anhydride liquid plugs was evaluated to determine the post-service unsealing window after thermal curing. Cured cylindrical specimens were first prepared under the selected curing conditions and cut into samples of similar size and mass. The initial mass of each dried specimen was recorded as m0 before degradation testing.

The degradation experiments were carried out by immersing the cured plug samples in the GVL/p-TSA degradation medium with a mass ratio of 95/5 (*w*/*w*) at 120, 130, and 140 °C. The liquid-to-solid mass ratio was kept constant for each test to ensure sufficient contact between the degradation medium and the polymer network. At predetermined time intervals, the samples were removed, gently rinsed with ethanol to eliminate residual degradation liquid, dried at 60 °C to constant mass, and weighed. The mass retention ratio was calculated using Equation (4):

(4) $$R_{m}=\frac{m_{t}}{m_{0}}\times 100\%$$

where *R_m_* is the mass retention ratio, *m*_0_ is the initial dry mass of the cured plug, and *m_t_* is the residual dry mass after degradation for time *t*. The degradation time, *t_d_*, was defined as the time required for the sample to lose its structural integrity or reach a stable low mass-retention state. Each condition was tested in triplicate, and the average value with standard deviation was reported.

### 3.12. Residual Mechanical Strength After Degradation

Residual compressive strength after degradation was measured to determine whether the degradation-induced mass loss and morphological collapse corresponded to loss of load-bearing capability. Cured specimens were degraded at 120, 130, and 140 °C according to the procedure described in Section 3.11. At selected degradation stages, corresponding to normalized degradation times of *t*/*t*_d_ = 0, 0.25, 0.50, 0.75, and 1.00, specimens were removed from the degradation medium.

The collected specimens were gently rinsed with ethanol to remove residual degradation medium and then dried at 60 °C until no visible surface liquid remained. The partially degraded specimens were subjected to uniaxial compression testing using the same universal testing machine and loading rate described in Section 3.7. The residual compressive strength was calculated from the maximum load divided by the initial cross-sectional area of the specimen. For each temperature and degradation stage, at least three parallel specimens were tested, and the results were reported as mean ± SD.

The post-service unsealing time *t*_d_ was defined as the time at which the specimen lost macroscopic structural integrity and retained only limited residual compressive strength. This definition allowed the unsealing window to be evaluated from both morphological and mechanical perspectives.

### 3.13. Construction of the Curing–Degradation Window Model

The curing–degradation window model was constructed by integrating the characteristic time parameters obtained from rheological, curing, and degradation measurements. Three key parameters were used to describe the time-programmed behavior of the ESO-based epoxy–anhydride liquid plug: the pumpable time (*t*_p_), curing time (*t*_c_), and post-service unsealing time (*t*_d_). Here, *t*_p_ was determined from the viscosity–time curve as the period during which the apparent viscosity remained below 100 mPa·s; tc was obtained from the inversion curing test; and td was recorded as the time required for the cured plug to lose its macroscopic structural integrity during degradation.

Based on these parameters, the material response was divided into three sequential stages: pumpable placement, thermally triggered curing, and post-service degradation. The relationship among *t*_p_, *t*_c_, and *t*_d_ was used to evaluate whether the precursor could remain injectable before placement, form a load-bearing network under high-temperature conditions, and subsequently undergo controlled structural disassembly after service. The resulting model was used as a quantitative framework to compare different temperature and formulation conditions and to identify a suitable sealing–unsealing window for high-temperature temporary sealing applications.

### 3.14. Statistical Analysis

All quantitative experiments were performed in triplicate unless otherwise stated. The results are expressed as mean ± standard deviation (SD). Statistical analysis was performed using one-way analysis of variance (ANOVA), followed by Tukey’s post hoc test for multiple comparisons when applicable. Differences were considered statistically significant at *p* < 0.05. For kinetic calculations based on DSC data, the reported values were derived from the corresponding non-isothermal DSC curves and fitted according to the Kissinger, Ozawa, and Friedman methods. Graphs were plotted using OriginPro 2024b software (OriginLab Corporation, Northampton, MA, USA), and error bars in the figures represent SD.

## 4. Conclusions

In this study, an epoxidized soybean oil (ESO)-based epoxy–anhydride liquid plug was developed to construct a thermally regulated curing–degradation window for sustainable high-temperature temporary sealing. The optimized precursor, composed of E51 epoxy resin, methylhexahydrophthalic anhydride, n-butyl glycidyl ether, and ESO, exhibited a low initial viscosity of 65.4 ± 2.1 mPa·s and maintained a pumpable state below 100 mPa·s before thermal curing. By adjusting temperature and initiator dosage, the curing time could be regulated within 1–10 h at 120–140 °C, while DSC analysis further confirmed that the curing process was governed by temperature-accelerated epoxy–anhydride ring-opening and esterification reactions. The cured network showed a compressive strength of 112.5 ± 3.5 MPa and an elastic modulus of 142.50 ± 5.26 MPa at the optimized ESO dosage, demonstrating that bio-based modification did not compromise the load-bearing capacity. DMA, gel fraction, and swelling results revealed that ESO improved the stiffness–flexibility balance by introducing reactive flexible segments into the crosslinked network, whereas excessive ESO slightly reduced network compactness. FTIR analysis verified the consumption of epoxy and anhydride groups and the formation of ester-rich structures, supporting the reactive incorporation of ESO. During post-service degradation, the mass retention decreased in a temperature-dependent manner, and the characteristic unsealing time shortened from 84 h at 120 °C to 24 h at 130 °C and 18 h at 140 °C. Residual-strength measurements further showed that degradation was accompanied by a rapid loss of load-bearing capability, confirming *t*_d_ as both a morphological and mechanical failure time. These results establish an experimentally defined *t*_p_–*t*_c_–*t*_d_ window model that links pumpable placement, thermally triggered network formation, load-bearing sealing, and post-service unsealing within one bio-based epoxy–anhydride material platform. Compared with previously reported ESO-based plugging agents, this work provides a more explicit molecular–temporal regulation strategy by connecting ESO-mediated ester-rich network formation with staged pumpability, curing, mechanical sealing, and degradation-induced loss of load-bearing capacity. Nevertheless, the present study still has several limitations. The sealing-related performance was mainly evaluated through precursor pumpability, curing behavior, compressive strength, residual mechanical strength, mass-retention evolution, and post-service structural disassembly. Further sealing-performance experiments under flow-through conditions, pressure-driven leakage paths, or field-like wellbore environments were not conducted due to laboratory constraints and should be addressed in future work. In addition, although ESO was introduced as a bio-based reactive component and the cured plug showed controlled post-service degradability, a complete life-cycle assessment (LCA) was not performed in this study. The current sustainability discussion is therefore limited to material origin, degradability, and removability. A quantitative LCA will require complete inventory data for raw-material production, precursor preparation, curing energy consumption, degradation-medium use, waste-liquid treatment, and scale-up operation. Future work should combine core-flow sealing validation with LCA-based comparison against conventional petroleum-derived epoxy or non-degradable sealing systems to more comprehensively evaluate the practical and environmental benefits of this liquid plug platform.

## Data Availability

The original contributions presented in this study are included in the article. Further inquiries can be directed to the corresponding author.
